# Healthcare Worker Seroconversion in SARS Outbreak

**DOI:** 10.3201/eid1002.030397

**Published:** 2004-02

**Authors:** Pierce K. H. Chow, Eng-Eong Ooi, Hiang-Khoon Tan, Kong-Wee Ong, Bijon Kumar Sil, Melissa Teo, Timothy Ng, Khee-Chee Soo

**Affiliations:** * Singapore General Hospital, Singapore; † National Environment Agency, Singapore; ‡ Genome Institute of Singapore, Singapore

**Keywords:** severe acute respiratory syndrome, healthcare workers, outbreak, seroconversion, subclinical infection, dispatch

## Abstract

Serum samples were obtained from healthcare workers 5 weeks after exposure to an outbreak of severe acute respiratory syndrome *(*SARS). A sensitive dot blot enzyme-linked immunosorbent assay, complemented by a specific neutralization test, shows that only persons in whom probable SARS was diagnosed had specific antibodies and suggests that subclinical SARS is not an important feature of the disease**.**

## The Study

Severe acute respiratory syndrome (SARS) emerged only in late 2002, but the rapid transmission of the disease worldwide within a few months has led to serious public health concerns. The putative agent of this new disease, identified in March 2003, is a novel and more pathogenic strain of the commonly occurring coronavirus (*1*,*2*). Cases were initially defined according to syndrome features in the absence of diagnostic tests (*3*). Knowledge of the epidemiology of SARS remains incomplete (*4*).

The proportion of persons infected with SARS-associated coronavirus (SARS-CoV) whose infection remained subclinical is not known. Such information is important, not only to facilitate understanding of the virulence of the virus but, more importantly to determine whether the control measures currently employed are sufficient to halt the spread of the virus. Should asymptomatic infection occur in substantial numbers, the virus may continue to spread, despite the isolation of the clinically apparent cases; however, this would result in the more rapid development of herd immunity in the community. The aim of this study was to determine the seroprevalence of anti–SARS-CoV antibodies in a population of exposed healthcare workers who worked in wards where an outbreak occurred.

At the beginning of April 2003, an outbreak of SARS (diagnosed according to prevailing World Health Organization guidelines) occurred in the surgical wards of the Singapore General Hospital. The source was initially unknown, and all staff and patients in these wards were potentially exposed and were themselves potential sources of the SARS virus. To contain the spread, healthcare workers from these wards were either quarantined in their homes for 2 weeks or sequestered with the patients and continued to look after them, adopting full reverse-barrier practices (*5*).

Subsequent contact tracing pointed to an index case-patient, whose infection led to 38 cases of SARS (in healthcare workers, patients, and visitors) in these wards and to another 12 cases of SARS in the rest of the hospital campus before the outbreak was brought under control 3 weeks later. Of the 200 healthcare workers in the surgical wards quarantined or sequestered, SARS developed in 17, and milder symptoms developed in a number of others, which did not qualify for a diagnosis of SARS under prevailing WHO guidelines (*3*).

The study was approved by the Ethics Committee of the Singapore General Hospital. All 200 healthcare workers, comprising doctors, nurses, health attendants, and receptionists in these surgical wards who were quarantined after the initial outbreak, were invited to participate. A total of 87 people volunteered. Of these, three had a history of probable SARS but had recovered sufficiently to return to work. Another group of 12 house officers, who joined the department during the week the study started, were invited to participate as negative controls because they had no prior exposure to known SARS patients. Informed consent was obtained from those who wished to take part. Participants filled out a questionnaire about symptoms experienced during the preceding weeks and donated a sample of blood by venipuncture; the serum specimen was stored at –80°C until use. Immunoglobulin (Ig) G antibodies to SARS-CoV were detected by using a dot blot enzyme-linked immunosorbent assay (ELISA) using a culture- derived, heat-inactivated virus antigen (E-E Ooi, unpub. data) at a serum dilution of 1:100. When compared to results of an indirect immunofluorescent assay in a limited study comprising 32 case-patients with clinically diagnosed SARS and 977 control serum samples collected before the SARS outbreak, sensitivity and specificity were 100% and 99.8%, respectively. Samples that tested positive for IgG antibodies to SARS-CoV were further assayed for neutralizing antibodies by using the 50% tissue infective dose (TCID_50_) method, similar to that previously described (*6*), under biosafety level 3 conditions, in serial twofold dilution, ranging from 1:10 to 1:320. The virus isolate used in this study, SARS-CoV 2003VA2774, has been previously sequenced (*7*) and was isolated from a patient in whom SARS was diagnosed. All assays were carried out in duplicate, and positive serum controls, obtained from a volunteer convalescent-phase SARS patient, were included in every run.

Four samples tested strongly positive by dot blot ELISA, although only three of these were positive for neutralizing antibodies with titers of 1:60, 1:60, and 1:320. All three were volunteers in whom probable SARS was diagnosed. Nine other samples tested weakly positive by the dot blot ELISA, although these samples were all negative by neutralization test. Analysis of data provided by the questionnaire showed that of the 84 exposed persons in whom SARS did not develop, 32 had combinations of various symptoms. None of them had positive chest x-ray findings.

## Discussion

This is the first study to examine the seroprevalence of anti–SARS-CoV antibodies in a population with a high likelihood of having been exposed to the virus. The results indicate that all samples positive for neutralizing antibodies were from persons who had symptoms indicative of SARS (Table). None of the healthcare workers studied showed serologic evidence of subclinical infection. This result strongly validates the current infection control measures to contain the spread of this virus, i.e., early identification and isolation of case-patients.

**Table:** Symptoms of SARS-exposed healthcare workers exposed to severe acute respiratory syndrome^a^

*Symptoms*	*No. of persons*
Asymptomatic	52
Systemic^b^	28
Upper respiratory tract^c^	25
Respiratory^d^	15
Gastrointestinal tract^e^	10
Musculoskeletal^f^	15

^a^Of the 887 volunteers, 37 had symptoms that were not sufficient to qualify as having probable severe acute respiratory syndrome (SARS). None of the 32 had positive chest x-ray signs. ^b^Systemic symptoms: fever, malaise, lethargy, headache. ^c^Upper respiratory: symptoms: runny nose, sore throat, sore mouth or gums. ^d^Respiratory symptoms: cough, breathlessness, chest pain. ^e^Gastrointestinal tract symptoms: vomiting, diarrhea, abdominal colic. ^f^Musculoskeletal symptoms: muscle ache, joint aches.

The finding of dot blot–positive, but neutralizing antibody–negative, specimens could be due to several factors. We had chosen to screen the serum specimens at a low dilution to increase their sensitivity, which would then be confirmed by the serum neutralization test. False-positive reactions to the screening test is thus expected. Furthermore, these dot blot–positive specimens could be due to cross-reaction with other coronaviruses (*7*). Although negative findings in a small population are difficult to generalize, our results suggest that subclinical infection is not an important feature of SARS. We are currently conducting larger population studies to further investigate this finding.

In conclusion, in a population of healthcare workers who worked in surgical wards at the time of the outbreak, only those who sought treatment for probable SARS had anti–SARS-CoV antibodies, suggesting no subclinical infection. Early identification and isolation of cases are thus effective infection control methods.
